# Cross-sectional study of HIV living people cohort to specify indications to antiretroviral treatment in naïve patients

**DOI:** 10.1186/1758-2652-13-S4-P23

**Published:** 2010-11-08

**Authors:** AJ Pronin, GD Kaminskiy

**Affiliations:** 1 Moscow Regional AIDS Centre, Schepkina 61/2 build.8, Moscow, Russian Federation

## Purpose of the study

To determine groups in HIV living people cohort in the aspect of further HAART.

## Methods

The data on 2271 patients was investigated in a cross-sectional study. All patients at the moment of survey had no attributes of clinical progression of disease and did not receive HAART. Clinical exam and blood sampling was performed, in blood specimens HIV virus load (PCR m2000rt Abbott Biosystems analyzer, RealTime HIV-1 sets) and CD3+CD4+ and CD3+CD4- phenotypes of T-lymphocytes were analyzed (flow cytometer BD FACSCount, sets of antibodies TriTEST CD3/CD4/CD45). For 363 patients additionally CD3+CD8+ and CD3+CD4-CD8- (double negative) phenotypes of T-lymphocytes were determined. Duration of HIV infection and the age of the patient was also included into analysis. Received data was processed statistically by means of SPSS software.

## Summary of results

**Figure 1:**
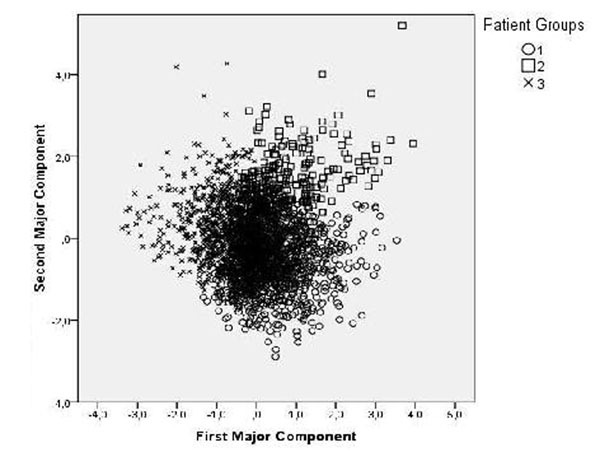
Bland-Altman plots comparing Framingham, Rama-EGAT, and D:A:D risk scores

Factor analysis with the principle component method revealed two major factors gathering 54% of the total variance. The first factor included CD4+ and CD3+CD4- T-lymphocyte counts with reverse influence of the viral load. The second factor included CD3+CD4- counts concordant with the viral load. Hierarchical cluster analysis by Ward method was performed in the space of these two factors and revealed 3 groups. Figure 1

The First Group had relatively small duration of disease (about 3 years), well preserved CD4 counts (650 cells/mm^3^), CD3+CD8+, CD3+CD4-CD8- T-lymphocytes on the average level characteristic for the HIV-living cohort. The group was designated as temporary no progressive. The Second Group had significant duration of disease (5 years), almost intact CD4 counts, 4,1 time increase in CD3+CD8+ T-lymphocytes and 10,3 increase of CD3+CD4-CD8- counts in comparison with healthy controls. This was permanent no progressive group. The Third Group had also significant duration of disease (6 years), different (from moderate to severe) depletion of all detected subpopulations of T-cells as well as the highest viral load. This was the progressive group, the members of which had indications to HAART (27% of the population). Table 1

**Table 1:** 

Group Number	Group Title	CD3+ (cells/mm^3^)	CD4 +(cells/mm^3^)	CD8 +(cells/mm^3^)	CD3+CD4-CD8- (cells/mm^3^)	Viral Load (log 10 copies/ml)	Age (years)	Disease Duration (years)
1	Temporary No Progressors	2321	654	1105	411	3,42	27,76	2,80

2	Permanently No Progressors	3575	735	2243	658	4,00	36,06	4,76

3	Progressors	1771	366	969	268	4,50	32,64	6,17

4	HIV Infected Total	2363	589	1195	394	3,80	30,34	4,01

5	Healthy Controls	1554	944	545	64	-	28,62	-

## Conclusions

1. Evaluation of no progressive status (temporary or permanent) as well as diagnostics of the progressive state outlines indications to antiretroviral treatment. 2. Evaluated internal groups support approach to start HAART after CD4 cell counts are less than 500/mm^3^ and the depletion level of 350 CD4 cells/mm^3^ lies in the middle scope of progressive group.

## References

[B1] Timing of initiation of antiretroviral therapy in AIDS-free HIV-1-infected patients: a collaborative analysis of 18 HIV cohort studies Lancet 2009 13 9672 1352 1363 April 18 10.1016/S0140-6736(09)60612-7 PMC2670965 19361855

[B2] O'Connell KA Bailey JR Blankson JN Elucidating the elite: mechanisms of control in HIV-1 infection Trends Pharmacol Sci 2009 13 12 631 7 Dec 10.1016/j.tips.2009.09.005 19837464

